# Subacute Pulmonary Embolism: Insights from a National Registry of Catheter-Directed Interventions

**DOI:** 10.3390/jcm15187135

**Published:** 2026-09-14

**Authors:** Alejandro Lara-García, Daniel Tébar Márquez, Carlos Real, Pablo Salinas-Sanguino, Gonzalo García-Martí, Francesco Costa, Belén Cid, Ignacio J. Amat-Santos, Javier Martín-Moreiras, Alfonso Jurado-Román, Raúl Moreno, on behalf of the ACI-SEC Pulmonary Embolism Registry Investigators

**Affiliations:** 1 Interventional Cardiology Unit, Hospital Universitario La Paz, Paseo de la Castellana 261, 28046 Madrid, Spain; daniel.tebar.m@gmail.com (D.T.M.); alfonsojuradoroman@gmail.com (A.J.-R.);; 2 Department of Cardiology, Institut Universitaire de Cardiologie et de Pneumologie de Québec (IUCPQ–UL), Laval University, Quebec City, QC G1V 4G5, Canada; carlosrealj42@gmail.com; 3 Interventional Cardiology Unit, Hospital Clínico San Carlos, 28040 Madrid, Spaingonzalete97@gmail.com (G.G.-M.); 4 Interventional Cardiology Unit, Hospital Universitario Virgen de la Victoria, 29010 Malaga, Spain; francesco.costa@unime.it; 5 Department of Cardiology, Hospital Clínico Universitario de Santiago de Compostela, 15706 Santiago de Compostela, Spain; 6 Department of Cardiology, Instituto de Ciencias del Corazón (ICICOR), Hospital Clínico Universitario de Valladolid, 47003 Valladolid, Spain; 7 Department of Cardiology, Instituto de Investigación Biomédica de Salamanca (IBSAL), Complejo Asistencial Universitario de Salamanca, 37007 Salamanca, Spain

**Keywords:** pulmonary embolism, subacute pulmonary embolism, catheter-directed therapy, mechanical thrombectomy, right ventricular dysfunction, pulmonary hypertension, cardiac biomarkers, NT-proBNP, troponin, registry

## Abstract

**Background:** Pulmonary embolism (PE) has classically been divided, on the basis of symptom duration, into acute (<2 weeks), subacute (2–12 weeks) and chronic (>12 weeks) forms. Subacute PE remains poorly characterised, is frequently underdiagnosed, and is largely absent from randomised trials and dedicated guideline recommendations. Whether the efficacy and safety of catheter-directed therapy (CDT) extend to subacute presentations, in which the thrombus is older, more organised and potentially more adherent, is unknown. We aimed to describe the clinical profile, management and early outcomes of patients with subacute PE selected for CDT within a national interventional registry. **Methods**: We analysed the National Registry of Catheter-Directed Interventions for the Management of Acute PE (NCT06348459), an investigator-initiated, multicentre registry promoted by the Interventional Cardiology Association of the Spanish Society of Cardiology (ACI-SEC). Subacute PE was defined as a symptom duration >14 days and <12 weeks. Baseline characteristics, echocardiographic variables, biomarkers, invasive pulmonary artery pressures, procedural data and early outcomes were described for the subacute group and set alongside the acute presentations treated in the same cohort. All between-group comparisons are descriptive and exploratory and are not intended to characterise subacute PE beyond this selected population. Continuous variables are median [interquartile range]. **Results**: Among 447 PE cases treated with catheter-directed therapy, 11 (2.5%) met the criteria for subacute PE and 436 (97.5%) for acute PE. Subacute patients presented with progressive dyspnoea (73%) over a median symptom duration of 21 [18–28] days and infrequent syncope (9%); two patients (18%) fulfilled ESC criteria for high-risk PE and three (27%) required vasoactive amines. Subacute patients showed a right ventricular (RV) basal diameter of 50 [48–52] mm (echocardiographic data available in 7 of 11) and an echocardiographic pulmonary artery systolic pressure of 60 [46–60] mmHg, with a high-sensitivity troponin I of 455 [85–767] ng/L and an NT-proBNP of 1433 [256–1970] pg/mL; corresponding values in acute PE were 47 [44–50] mm, 50 [43–60] mmHg, 237 [97–853] ng/L and 2200 [626–5535] pg/mL. None of the between-group differences reached statistical significance. Catheter-directed therapy achieved 100% procedural success in the subacute group, with invasive systolic pulmonary artery pressure falling from 58 [53–60] to 48 [39–51] mmHg and peripheral oxygen saturation improving from 92% [89–95] to 95% [92–98], with no major periprocedural complications, no circulatory support and no in-hospital deaths. **Conclusions**: Subacute PE was an infrequent but clinically recognisable presentation among patients treated with catheter-directed therapy, accounting for 2.5% of the cohort and characterised by weeks of progressive symptoms before referral and by a substantial right ventricular and pulmonary pressure load at presentation. Mechanical thrombectomy was safe and effective in this subgroup, with favourable early outcomes comparable to those of acute PE. These hypothesis-generating findings support dedicated study of the diagnosis, risk stratification and interventional management of subacute PE.

## 1. Introduction

Acute pulmonary embolism (PE) is the third most frequent cardiovascular cause of death and a major source of morbidity worldwide. Contemporary management rests on early risk stratification, integrating haemodynamic status, imaging evidence of right ventricular (RV) pressure overload and/or RV dysfunction, cardiac biomarkers, and validated clinical scores, as set out in the 2019 European Society of Cardiology (ESC) guidelines [1]. RV pressure overload frequently precedes the development of overt RV dysfunction and is in itself a central component of the pathophysiology of PE. This framework, however, has been developed around the acute presentation of the disease. Both the ESC guidelines and the American Heart Association scientific statement on interventional therapies are explicitly restricted to acute PE [1,2], and the pivotal trials of reperfusion on which contemporary practice rests have restricted enrolment to patients within 14 to 15 days of symptom onset [3,4,5,6,7]. Patients whose symptoms have lasted longer are therefore absent from this evidence base by design.

From a temporal standpoint, PE has classically been arbitrarily divided according to symptom duration into acute (<2 weeks), subacute (2–12 weeks) and chronic (>12 weeks) forms [8]. While the acute and chronic ends of this spectrum are comparatively well described, subacute PE occupies an ambiguous intermediate territory: it remains poorly characterised, is frequently underdiagnosed, and is largely absent from randomised trials and dedicated guideline recommendations. Patients with a more prolonged symptom course may have undergone a period of progressive pulmonary vascular obstruction with concomitant RV adaptation and remodelling [9,10]. Sustained RV pressure overload promotes maladaptive RV remodelling and may represent an intermediate step towards chronic thromboembolic pulmonary hypertension (CTEPH), which develops in approximately 2–3% of PE survivors and carries substantial morbidity and mortality [11,12].

Over the past decade, catheter-directed therapies, and in particular large-bore mechanical thrombectomy, have emerged as effective options for selected patients with intermediate–high-risk and high-risk acute PE, with favourable safety and haemodynamic results in trials and registries such as FLARE, FLASH, and PEERLESS [6,7,13]. Risk stratification and the choice of reperfusion modality are, however, conceptually distinct decisions: symptom duration is not a component of any validated risk score, nor of the criteria that establish the indication for reperfusion, and we do not propose that it should be. Reperfusion, in contrast, must act upon the thrombus itself, and thrombus composition is time-dependent: over days to weeks an erythrocyte- and fibrin-rich clot undergoes fibrin homogenisation, leukocyte degeneration, fibroblast ingrowth and surface endothelialisation, becoming firmer, more organised and progressively more adherent to the vessel wall [14,15]. These changes have measurable functional correlates. In a pooled analysis of 308 patients from five thrombolysis trials, the degree of reperfusion achieved fell by 0.8% of lung tissue for each additional day of symptoms before treatment, and angiographic clot lysis was lower in patients with symptoms of at least six days than in those with symptoms of one day or less, although thrombolysis remained useful in patients treated up to 14 days after symptom onset [16]. Experimentally, the accessibility of the fibrin network to circulating plasminogen activators declines as a venous thrombus ages and predicts fibrinolytic efficacy [17], and the histological composition of an embolus determines the performance of mechanical thrombectomy devices, albeit demonstrated in cerebral circulation [18]. Whether these properties translate into a reduced efficacy of large-bore aspiration in the pulmonary arteries has not been established.

Accordingly, many operators regard a prolonged symptom duration as a relative, or even absolute, contraindication to catheter-directed treatment, anticipating a longer, technically more demanding and less effective procedure. This expectation rests on pathophysiological inference rather than on outcome data, and it generates a self-perpetuating evidence gap: these patients are less often offered intervention, the resulting series remain small, and the assumption remains untested. Preliminary single-centre experience from our group suggested that mechanical thrombectomy can effectively remove the older, organised thrombus characteristic of subacute PE [19,20], but this has not been examined at a national, multicentre level. Against this background, the present study had two descriptive objectives, both explicitly restricted to patients selected for catheter-directed intervention: (1) to characterise the clinical presentation, echocardiographic findings and biomarker profile of patients with subacute PE at the time of referral for intervention, alongside those of the acute presentations treated within the same cohort; and (2) to describe the procedural results, safety and early in-hospital outcomes of catheter-directed mechanical thrombectomy in subacute presentations. We did not set out to establish whether subacute PE constitutes a distinct clinical entity: that question cannot be addressed in a population restricted to patients selected for intervention, and would require a consecutive, all-comer cohort of patients with pulmonary embolism. Accordingly, all between-group comparisons are presented as descriptive and exploratory, and no inference is intended regarding the phenotype of subacute PE beyond the population studied.

## 2. Materials and Methods

### 2.1. Study Design and Population

We performed a post hoc analysis of the National Registry of Catheter-Directed Interventions for the Management of Acute PE (ClinicalTrials.gov identifier NCT06348459), an investigator-initiated, multicentre, prospective registry promoted by the Interventional Cardiology Association of the Spanish Society of Cardiology (ACI-SEC). The registry was launched in 2018 and enrols patients with confirmed PE who are evaluated for, and treated with, catheter-directed therapy. Of the 118 participating centres, those with an active pulmonary embolism response team (PERT) and catheter-directed intervention programme contributed cases during the study period. All consecutive patients undergoing catheter-directed therapy for PE were included.

### 2.2. Definitions

Patients were classified according to symptom duration prior to diagnosis. In line with the classical temporal classification of PE [8], subacute PE was defined as a symptom duration greater than 14 days (>2 weeks) and less than 12 weeks; patients with a symptom duration ≥ 12 weeks (chronic presentations) were not represented in the treated cohort. All remaining patients were classified as acute PE. This definition therefore captured genuinely subacute presentations, excluding chronic thromboembolic disease. Risk stratification followed the 2019 ESC guidelines, integrating haemodynamic status, RV dysfunction on imaging, cardiac biomarkers and clinical scores (Pulmonary Embolism Severity Index [PESI]/simplified PESI and the BOVA score) [1,21,22]. High-risk PE was defined, in accordance with the 2019 ESC guidelines, by haemodynamic instability, that is, cardiac arrest, obstructive shock or persistent hypotension (systolic blood pressure < 90 mmHg, or vasopressors required to achieve a systolic blood pressure ≥ 90 mmHg despite adequate filling status, with end-organ hypoperfusion) [1]. The requirement for vasoactive amines and the Society for Cardiovascular Angiography and Interventions (SCAI) shock stage were recorded separately, so that haemodynamic status could be described beyond systemic blood pressure alone.

### 2.3. Treatment Strategy

The indication for catheter-directed therapy was established by the local PERT/Heart Team and included high-risk PE, intermediate–high-risk PE with a BOVA score ≥ 4, and PE with refractory respiratory failure, as well as patients with contraindications to or failure of systemic fibrinolysis. The same treatment algorithm was applied to acute and subacute presentations. Percutaneous mechanical thrombectomy was performed predominantly with large-bore aspiration systems (FlowTriever; Inari Medical, Irvine, CA, USA) and, in a minority of cases, with other aspiration devices (Indigo Aspiration System; Penumbra, Inc., Alameda, CA, USA, and other aspiration catheters).

### 2.4. Data Collection and Outcomes

Baseline clinical characteristics and haemodynamic status were collected, together with the echocardiographic variables recorded in the registry case report form: RV basal diameter (mm), left ventricular end-diastolic diameter (mm), the RV/left ventricular ratio, the presence of qualitative RV dysfunction, the presence of right heart thrombus, the degree of tricuspid regurgitation and the pulmonary artery systolic pressure (PASP, mmHg) estimated from the tricuspid regurgitation jet. Parameters such as TAPSE, tricuspid annular systolic velocity, and RV strain were not captured in the national case report form and were therefore not available. Cardiac biomarkers comprised high-sensitivity troponin I, expressed in ng/L and recorded as the peak value during the index admission, and NT-proBNP, expressed in pg/mL and recorded at admission. Both biomarker fields are completed retrospectively by each participating centre from the local clinical record and are not mandatory for case validation. Invasive systolic, diastolic and mean pulmonary artery pressures were measured immediately before and after the procedure. Pre-specified outcomes were procedural success, periprocedural haemodynamic improvement (invasive pulmonary artery pressure and peripheral oxygen saturation), major and minor complications, need for circulatory support, and in-hospital survival.

### 2.5. Statistical Analysis

Continuous variables are expressed as the median with the interquartile range (IQR), given in parentheses in the running text and in square brackets in the Abstract and tables, and compared between groups with the Mann–Whitney U test; categorical variables are expressed as counts and percentages and compared with the χ^2^ or Fisher exact test, as appropriate. A two-sided *p*-value < 0.05 was considered statistically significant. Given the small number of subacute cases (*n* = 11), all between-group comparisons are exploratory and hypothesis-generating, and were not adjusted for multiplicity. Analyses were performed in Python v3.11 (Python Software Foundation, Beaverton, OR, USA) with the pandas (v2.2) and SciPy (v1.14) libraries.

### 2.6. Ethics

The registry protocol was approved by the Clinical Research Ethics Committee (CEIm) of Hospital Clínico San Carlos, Madrid, Spain, which acted as the central ethics committee for all participating Spanish centres (protocol code 18/010-E; approved 18 September 2018). The study was conducted in accordance with the Declaration of Helsinki. All participants provided written informed consent.

## 3. Results

### 3.1. Prevalence and Clinical Presentation

Among the 447 PE cases treated with catheter-directed therapy, 11 (2.5%) fulfilled criteria for subacute PE and 436 (97.5%) for acute PE. Subacute presentations were therefore infrequent but clinically recognisable, with symptom duration ranging from 17 to 33 days and no chronic cases. Patients with subacute PE typically reported several weeks of progressive dyspnoea (73%) followed by recent clinical deterioration, with a median symptom duration of 21 days (IQR 18–28), rather than sudden onset. Syncope at presentation was uncommon (one patient, 9%). Haemodynamic status at admission was heterogeneous and is not adequately summarised by systemic blood pressure alone. Although the median systolic blood pressure was 110 mmHg (IQR 100–122), two patients (18%) fulfilled ESC criteria for high-risk PE and the remaining nine (82%) were classified as intermediate–high risk; three patients (27%) required vasoactive amines and were therefore classified as SCAI shock stage C, four (36%) as stage B and four (36%) as stage A. Patient-level clinical, haemodynamic and procedural data for the 11 subacute cases are provided in Supplementary Table S1 (Table 1).

### 3.2. Echocardiographic and Invasive Haemodynamic Findings

Transthoracic echocardiography was available in 7 of the 11 subacute patients. In these patients the median RV basal diameter was 50 mm (IQR 48–52) and the median estimated PASP 60 mmHg (IQR 46–60); the corresponding median values in acute PE were 47 mm (IQR 44–50) and 50 mmHg (IQR 43–60). Neither difference reached statistical significance (*p* = 0.10 and *p* = 0.52, respectively) and, given the number of subacute patients with available echocardiographic data, no comparative inference is drawn from these values. Invasive pulmonary artery pressures measured at the time of the procedure were available in 10 subacute patients: median systolic 58 mmHg (IQR 53–60), median diastolic 18 mmHg (IQR 14–24), and median mean pressure 32 mmHg (IQR 28–37), compared with median values of 53 mmHg (IQR 45–65), 22 mmHg (IQR 17–26), and 33 mmHg (IQR 28–40), respectively, in acute PE (Table 1).

### 3.3. Biomarker Profile

The median peak high-sensitivity troponin I was 455 ng/L (IQR 85–767) in subacute PE and 237 ng/L (IQR 97–853) in acute PE (*p* = 0.86), and the median NT-proBNP 1433 pg/mL (IQR 256–1970) and 2200 pg/mL (IQR 626–5535), respectively (*p* = 0.24). A cardiac troponin determination was recorded in 10 of the 11 subacute patients (91%) and in 421 of the 436 acute patients (97%); the peak value, which constitutes a separate and non-mandatory field of the case report form, was available in nine subacute (82%) and 297 acute patients (68%), and NT-proBNP in 9 (82%) and 341 (78%), respectively. Missing biomarker data were therefore not attributable to the biomarker not having been measured and did not track clinical severity: the peak troponin was available in 70% of patients meeting ESC criteria for high-risk PE versus 68% of the remainder, and NT-proBNP in 75% versus 81%. Neither difference was statistically significant and the interquartile ranges overlapped substantially. These values are therefore reported as descriptive data for the two groups treated within the registry rather than as evidence of a differing biomarker profile, and the direction of the difference is considered further in the Discussion (Table 1).

### 3.4. Procedural Data and Early Outcomes

All subacute patients underwent catheter-based mechanical thrombectomy because of high-risk or intermediate–high-risk profiles and contraindications to, or insufficient response to, standard therapy. Large-bore aspiration thrombectomy with the FlowTriever system was used in the majority of subacute cases (7 of 11), with other aspiration devices used in the remainder. Procedural (technical) success, understood as effective thrombus removal with consequent respiratory and haemodynamic improvement, was achieved in all subacute patients. Catheter-directed therapy produced rapid haemodynamic and respiratory improvement: the median invasive systolic pulmonary artery pressure fell from 58 mmHg (IQR 53–60) to 48 mmHg (IQR 39–51) and the median mean pulmonary artery pressure from 32 mmHg (IQR 28–37) to 27 mmHg (IQR 21–29), while the median peripheral oxygen saturation improved from 92% (IQR 89–95) to 95% (IQR 92–98). In the three subacute patients in whom echocardiography was repeated before discharge, the median estimated PASP was 34 mmHg (IQR 29–48). No major periprocedural complications (procedure-related death, major bleeding, cardiac perforation or significant vascular access complications) were observed in the subacute group; no subacute patient required circulatory support, and there were no in-hospital deaths. In the acute group, major periprocedural complications were likewise infrequent (5.7%), although disease-related in-hospital mortality was relatively high (12%), taking into consideration that it was a population that included high-risk and cardiac-arrest presentations (Table 2).

### 3.5. Single-Centre Experience: The Highest-Recruiting PERT

**Figure 1:**
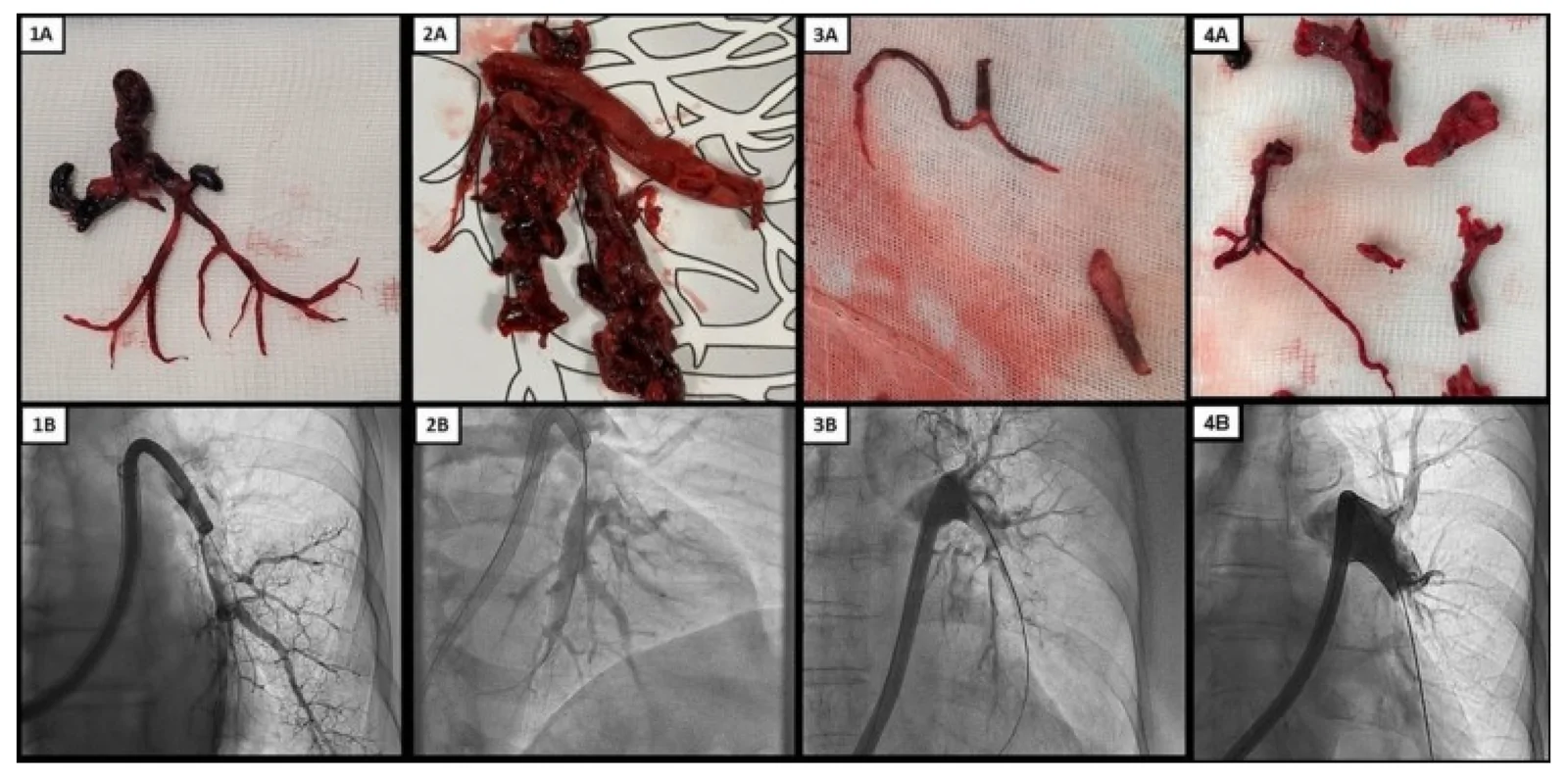
Representative subacute pulmonary embolism cases treated with catheter-directed mechanical thrombectomy at the highest-recruiting centre. Panels (**1A**–**4A**) show the organised subacute thrombus material retrieved by large-bore aspiration thrombectomy in four patients; panels (**1B**–**4B**) show the corresponding pulmonary angiograms. The abundant, organised nature of the extracted thrombi is consistent with a more prolonged, subacute time course.

To illustrate how subacute PE is managed within a mature, high-volume PERT programme, we describe the experience of the highest-recruiting subacute PE centre in the registry, which contributed 33 of the 447 catheter-treated PE cases and 6 of the 11 subacute cases, more than any other participating centre, and where catheter-directed therapy is delivered through a structured PERT protocol integrating risk stratification (BOVA score, biomarkers and echocardiographic RV assessment) with a predefined treatment algorithm applied uniformly to acute and subacute presentations. In this single-centre cohort of catheter-treated PE, the FlowTriever system was used in 96% of procedures, with procedural success in 100% and no rescue therapies required. Major complications were rare (2%, corresponding to periprocedural haemodynamic deterioration managed with VA-ECMO), with no access-site or remote vascular complications; the mean haemoglobin reduction was 1.3 g/dL and transfusion was required in 8%. Survival was 96%, and echocardiography at discharge showed a normal RV in 80% of cases. A learning-curve effect was observed: the median procedural time fell from approximately 85 min over the first five procedures to 60 min between the fifth and twentieth cases, and has since plateaued at approximately 50 min beyond the twentieth case. At a comparable point of the learning curve, subacute cases consistently required around 15 min more than acute ones. Representative cases of subacute thrombus retrieved by mechanical thrombectomy, with the corresponding pulmonary angiograms, are shown in Figure 1. These single-centre figures pertain to the overall catheter-treated PE cohort of this centre and are presented as contextual, real-world experience rather than as outcomes of the national subacute subgroup.

### 3.6. Histopathological Correlation of a Subacute Thrombus

**Figure 2:**
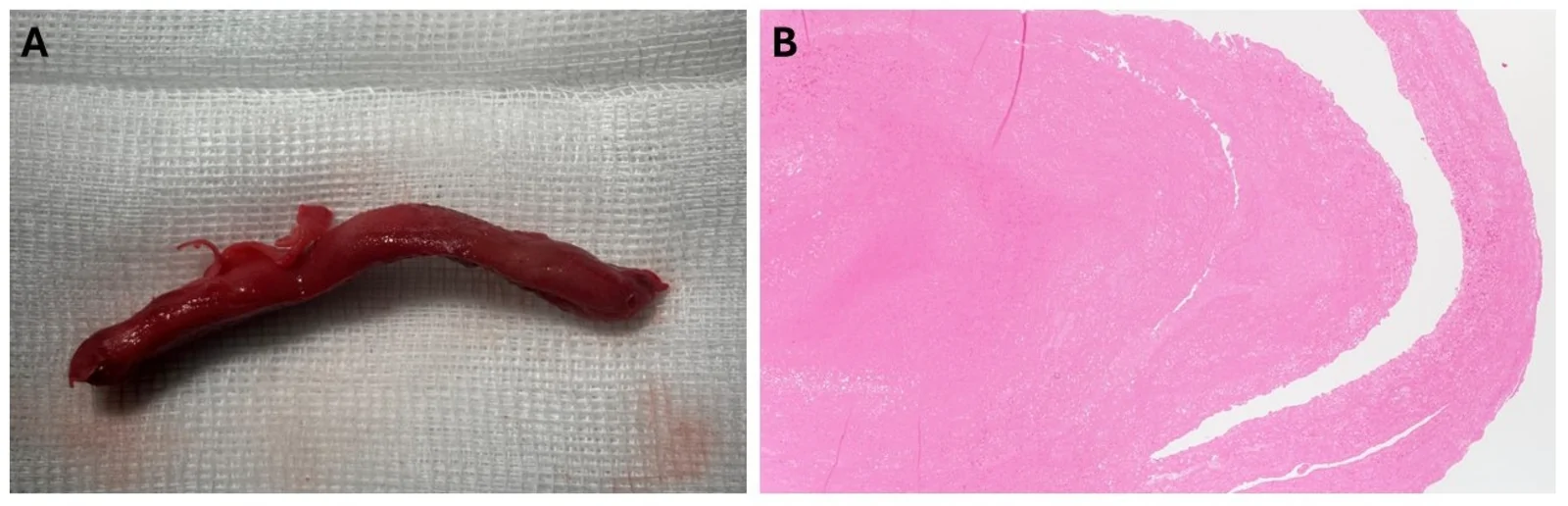
Histopathological correlation in a subacute pulmonary embolism case. (**A**) Macroscopic appearance of the thrombotic cast retrieved from the left main pulmonary artery by large-bore aspiration thrombectomy. (**B**) Corresponding haematoxylin–eosin section showing abundant, homogenised fibrin-rich material with features of organisation and few acute features. Organising (subacute) thrombi are characterised by homogenisation of fibrin, leukocyte degeneration, haemosiderin-laden macrophages, early fibroblast ingrowth with surface endothelialisation, and, at later stages, incipient neovascularisation and fibrous substitution, whereas acute features such as lines of Zahn and neutrophil pyknosis are correspondingly reduced [14,15].

In one subacute patient, the thrombotic material retrieved by large-bore aspiration thrombectomy from the left main pulmonary artery was submitted for histopathological analysis. Macroscopically it formed an elongated endoluminal cast; on haematoxylin–eosin examination it consisted of abundant, homogenised fibrin-rich material with morphological features of organisation and comparatively few acute features (Figure 2). This pattern, fibrin homogenisation and organisational remodelling with reduced acute changes, corresponds to the histological hallmarks that, according to established thrombus-dating criteria, differentiate older, organising (subacute) thrombi from acute ones [14,15]. Whereas hyperacute and acute thrombi are dominated by lines of Zahn, neutrophil pyknosis, and abundant trapped erythrocytes giving them their characteristic deep red colour, organising thrombi are progressively characterised by homogenisation of fibrin, degeneration and lysis of leukocytes, haemosiderin-laden macrophages, early fibroblast ingrowth with surface endothelialisation, and, at later stages, incipient neovascularisation and fibrous substitution, which will turn their colour to a palish pink [14,15]. The predominance of these organisational features, together with scant acute changes, is consistent with the more prolonged, subacute time course of this presentation. Notably, despite corresponding to an older, more organised and potentially more wall-adherent thrombus, the material was removed safely and effectively by percutaneous mechanical thrombectomy. These observations derive from a single retrieved specimen and are therefore illustrative and hypothesis-generating rather than representative of subacute thrombi in this cohort or in general.

## 4. Discussion

In this national registry of catheter-directed interventions, subacute PE accounted for only 2.5% of treated cases. The principal finding is therapeutic: catheter-directed mechanical thrombectomy achieved uniform procedural success, a reduction in invasive pulmonary artery pressure, and improved oxygenation in these patients, without major periprocedural complications, need for circulatory support, or in-hospital death. The clinical, echocardiographic and biomarker data are presented as a description of the subacute patients who reached the catheterisation laboratory. Because the sample was small, no comparison reached statistical significance and the population is by definition selected, they do not establish that subacute PE constitutes a distinct clinical entity, a question that would require a consecutive, all-comer cohort [8].

The clinical picture of progressive dyspnoea over approximately three weeks, with infrequent syncope, contrasts with the abrupt onset of many acute events and carries a real risk of diagnostic delay. It should not, however, be equated with haemodynamic stability: two of the 11 subacute patients met ESC criteria for high-risk PE and three required vasoactive amines. A normal or near-normal systemic blood pressure in this setting is an insufficient marker of RV jeopardy [1,23]; this caution applies with particular force when the insidious tempo of the illness may itself be reassuring to the treating clinician.

The biomarker values deserve a cautious reading, and the two markers reflect different processes. Cardiac troponin release in acute PE indicates myocardial injury of the pressure-overloaded right ventricle, in which increased wall tension and subendocardial ischaemia produce microscopic RV necrosis in the absence of epicardial coronary disease [24,25]. Its elevation identifies patients at higher risk of early death and adverse outcome [24] and is incorporated into the intermediate-risk stratum of current guidelines, particularly in combination with imaging evidence of RV dysfunction and clinical scores [1,26]. Natriuretic peptides, by contrast, are released by cardiomyocytes in response to myocardial wall stretch, so that their circulating concentrations track the pressure and volume load imposed on the ventricle: in pulmonary hypertension, plasma natriuretic peptide concentrations correlate with mean pulmonary artery pressure, pulmonary vascular resistance and right atrial pressure [27], and in acute PE, NT-proBNP correlates with right, but not left, ventricular volume and function [28] and predicts adverse outcome [29]. On mechanistic grounds, a subacute, pressure-overloaded RV that has had weeks to remodel might therefore be expected to show a relatively higher NT-proBNP, reflecting sustained wall stress, and a relatively lower troponin than an abrupt acute event in which myocardial injury is maximal at presentation [9,25,27]; the values observed here ran in the opposite direction. We do not interpret this as a biological signature of subacute disease, for three reasons. First, all differences were non-significant, with wide and overlapping interquartile ranges and very few subacute patients. Second, and most plausibly, the pattern reflects the comparator: the acute arm, itself selected for catheter-directed therapy, was enriched for massive, sudden, high-risk events, including cardiac-arrest presentations, in which natriuretic-peptide release is particularly marked, pulling the acute NT-proBNP distribution upwards, with an interquartile range extending beyond 5000 pg/mL. Third, patients whose presentation is insidious often do not seek medical attention early, and operators remain reluctant to intervene once symptoms have been prolonged; those who ultimately reach the catheterisation laboratory are therefore likely to represent the most decompensated end of the subacute spectrum, managed at high-complexity centres.

Two aspects of the troponin data deserve particular emphasis. First, high-sensitivity troponin I was clearly elevated in the subacute group, with a median of 455 ng/L and an interquartile range extending to 767 ng/L, and was above the upper reference limit in eight of the nine patients in whom it was recorded. Several weeks of symptoms therefore do not imply a quiescent or burnt-out process: these patients had ongoing right ventricular myocardial injury at the time of referral, a reading consistent with the observation that two of the 11 (18%) met ESC criteria for high-risk PE and three (27%) required vasoactive amines. This is of practical relevance, because the insidious tempo of a subacute presentation may otherwise be mistaken for stability, and because troponin is precisely the variable that current risk-stratification algorithms use to identify such patients [1,24,26]. Second, the registry recorded the peak troponin reached during the index admission rather than a value sampled at a protocolised time point, and the interval between symptom onset, admission, and sampling was neither standardised nor recorded. Since troponin follows a time-dependent release and clearance curve, an unstandardised peak cannot be compared across groups whose symptom duration differs by definition, and these values are best read as an indication that myocardial injury was present rather than as a quantitative comparison between the two presentations. Any biomarker characterisation of subacute PE will consequently require prospective, protocolised and paired sampling in an unselected population, and cannot be derived from data such as ours.

From a therapeutic standpoint, the second question was whether the favourable results of catheter-directed therapy in acute PE [6,13] extend to subacute disease, where the thrombus is older, more organised and potentially more adherent, and the RV more remodelled. In our experience, mechanical thrombectomy with large-bore aspiration devices achieved uniform procedural success, a reduction in invasive systolic pulmonary artery pressure from 58 to 48 mmHg, and improved oxygenation, with no major periprocedural complications, no need for circulatory support, and no in-hospital deaths in the subacute group. Consistent with the older nature of these thrombi, histopathological examination of the single specimen retrieved in one patient showed abundant fibrin with organisational remodelling and few acute features (Figure 2), in keeping with established thrombus-dating criteria [14,15]; nonetheless, this organised material was removed safely and effectively. This extends earlier single-centre observations from our group, in which subacute thrombi formed large, well-defined casts reproducing the pulmonary vascular architecture and were retrievable by mechanical thrombectomy, albeit over longer procedures [19,20]. These findings are consistent with the safety and efficacy reported in large thrombectomy registries [6,13] and suggest that, in selected subacute patients, catheter-directed treatment is both feasible and effective. It should be noted that the FLASH registry, the one large study that did not apply a symptom-duration criterion, did not record symptom duration either, so the number of subacute patients treated within it cannot be established.

It has been suggested that treating subacute PE might also prevent progression towards chronic thromboembolic disease and CTEPH, which complicate a minority of PE survivors but carry considerable long-term morbidity [11,12]. This hypothesis should be advanced with caution. In the long-term follow-up of the PEITHO trial, thrombolysis, despite achieving substantial early thrombus resolution, did not reduce the incidence of CTEPH or of persistent functional limitation compared with anticoagulation alone [30]. Effective early thrombus removal cannot therefore be assumed to translate into protection against chronic thromboembolic disease. Whether debulking of organised thrombus in subacute PE modifies long-term RV function, functional capacity, or the incidence of CTEPH remains an entirely open question—one that our data, limited to the in-hospital period, cannot address.

### Limitations

This study has several limitations. First and foremost, the number of subacute cases was very small (*n* = 11), so all comparisons are exploratory and underpowered; the non-significant *p*-values and wide interquartile ranges preclude firm inference, and the consistent directionality of some differences should be regarded as hypothesis-generating only. Second, and most importantly, the registry includes only patients selected for catheter-directed therapy. This is a doubly selected population: patients had to be considered candidates for reperfusion, and their treating team had to be willing to intervene despite a prolonged symptom duration, which many operators still regard as a relative contraindication. The subacute patients described here are therefore likely to represent the more severely affected end of the subacute spectrum, managed at high-complexity centres, and no characterisation of subacute PE as a clinical entity can legitimately be derived from them. That would require a consecutive, all-comer cohort of patients with pulmonary embolism. The same selection shapes the acute comparator, which is enriched for high-risk and cardiac-arrest presentations, and therefore every between-group comparison reported here. Third, the definition of subacute PE relies on patient-reported symptom duration, which is subject to recall bias. Fourth, outcomes were limited to the early in-hospital period, without systematic follow-up of RV recovery, functional status or CTEPH development. Fifth, echocardiographic data were available in only 7 of the 11 subacute patients and invasive pressures in 10, which further limits the precision of the values reported. Sixth, biomarker data were incomplete in both groups, reflecting retrospective, centre-level completion of non-mandatory case report form fields rather than a clinical decision not to measure them; although missingness was unrelated to ESC risk category, residual centre-level bias cannot be excluded.

## 5. Conclusions

In the Spanish national registry, subacute PE was an infrequent but clinically recognisable presentation among patients treated with catheter-directed therapy, accounting for 2.5% of the cohort and characterised by weeks of progressive symptoms before referral and by a substantial right ventricular and pulmonary pressure load at presentation. Catheter-directed mechanical thrombectomy was safe and effective in this subgroup, with early haemodynamic and clinical results comparable to those of acute PE. Because these observations derive from patients selected for intervention, they do not define the phenotype of subacute PE as a whole; however, they do support dedicated studies addressing the diagnosis, risk stratification, and interventional management of subacute PE.

## Figures and Tables

**Table 1:** Baseline characteristics of subacute versus acute pulmonary embolism. Continuous variables are median [interquartile range]; categorical variables are *n* (%).

Variable	Subacute PE (*n* = 11)	Acute PE (*n* = 436)	*p*-Value
Demographics			
Age, years	65 [62–74]	66 [54–74]	0.75
Female sex, n (%)	7 (64)	190 (44)	0.23
Body mass index, kg/m2	27 [25–37]	29 [26–33]	0.94
**Cardiovascular risk factors and comorbidity**			
Arterial hypertension, *n* (%)	7 (64)	220 (51)	0.54
Diabetes mellitus, *n* (%)	1 (9)	87 (20)	0.70
Current/former smoking, *n* (%)	3 (27)	147 (34)	0.76
Obesity, *n* (%)	5 (45)	171 (39)	0.92
Active cancer, *n* (%)	1 (9)	97 (22)	0.47
Previous pulmonary embolism, *n* (%)	0 (0)	37 (9)	0.61
Previous deep vein thrombosis, *n* (%)	1 (9)	56 (13)	1.00
Chronic lung disease (COPD), *n* (%)	2 (18)	28 (6)	0.16
Chronic kidney disease, *n* (%)	1 (9)	32 (7)	0.58
**Clinical presentation**			
Symptom duration, days	21 [18–28]	0 [0–2]	<0.001
Progressive dyspnoea, *n* (%)	8 (73)	254 (59)	0.54
Syncope at presentation, *n* (%)	1 (9)	88 (20)	0.70
Concomitant proximal DVT, *n* (%)	4 (40)	170 (40)	1.00
**Vital signs and severity**			
Systolic blood pressure, mmHg	110 [100–122]	110 [90–126]	0.48
Heart rate, bpm	105 [98–112]	110 [97–121]	0.45
Peripheral oxygen saturation, %	92 [89–95]	93 [89–96]	0.81
High-risk PE (ESC), n (%)	2 (18)	165 (38)	0.22
BOVA score	4 [4–5]	5 [4–7]	0.16
sPESI score	2 [1–3]	2 [1–3]	0.37
**Biomarkers**			
High-sensitivity troponin I, ng/L	455 [85–767]	237 [97–853]	0.86
NT-proBNP, pg/mL	1433 [256–1970]	2200 [626–5535]	0.24
**Echocardiography**			
RV basal diameter, mm	50 [48–52]	47 [44–50]	0.10
PASP, mmHg	60 [46–60]	50 [43–60]	0.52

COPD, chronic obstructive pulmonary disease; DVT, deep vein thrombosis; ESC, European Society of Cardiology; NT-proBNP, N-terminal pro–B-type natriuretic peptide; PASP, pulmonary artery systolic pressure; PE, pulmonary embolism; RV, right ventricular; sPESI, simplified Pulmonary Embolism Severity Index. Echocardiographic variables were available in 7 of the 11 subacute patients (RV basal diameter in 157 and PASP in 147 acute patients); high-sensitivity troponin I was available in 9 of 11 and NT-proBNP in 9 of 11 subacute patients. Bold are category headings and contain no data.

**Table 2:** Procedural characteristics and early in-hospital outcomes.

Variable	Subacute PE (*n* = 11)	Acute PE (*n* = 436)
FlowTriever system, *n* (%)	7 (64)	204 (60)
Procedural success, %	100	92
Invasive systolic PAP, pre → post, mmHg	58 → 48	53 → 40
Oxygen saturation, pre → post, %	92 → 95	93 → 96
Circulatory support (VA-ECMO), %	0	7
Major periprocedural complications, %	0	5.7%
In-hospital death, %	0	12

PAP, pulmonary artery pressure (invasive, measured immediately before and after the procedure); VA-ECMO, veno-arterial extracorporeal membrane oxygenation.

## Data Availability

The data that support the findings of this study are available from the ACI-SEC PE Registry on reasonable request and subject to registry governance approval.

## Supplementary Materials

The following supporting information can be downloaded at: https://www.mdpi.com/article/10.3390/jcm15187135/s1, Table S1: Clinical, haemodynamic and procedural characteristics of the 11 subacute pulmonary embolism patients (ordered by symptom duration).
