# Supplementary material for: Subacute Pulmonary Embolism: Insights from a National Registry of Catheter-Directed Interventions
Source: J Clin Med. 2026 Sep 14;15(18):7135. doi: 10.3390/jcm15187135 (PMC13607774; doi:10.3390/jcm15187135)
Supplement: Supplementary file 1 [file jcm-15-07135-s001.zip › jcm-4474561-supplementary.pdf]

Supplementary Material

Table S1. Clinical, haemodynamic and procedural characteristics of the 11 subacute pulmonary embolism patients (ordered by symptom duration).

Variable	P1	P2	P3	P4	P5	P6	P7	P8	P9	P10	P11
**Demographics**											
**Age, years**	79	77	65	78	60	63	64	70	66	38	56
**Sex**	M	M	F	F	M	F	F	F	M	F	F
**BMI, kg/m²**	26	27	19	25	36	25	38	23	43	37	35
**Cardiovascular risk factors / comorbidity**											
**Arterial hypertension**	Yes	No	No	Yes	Yes	No	Yes	Yes	Yes	No	Yes
**Diabetes mellitus**	No	No	No	No	No	No	No	No	No	No	Yes
**Smoking**	No	Yes	No	No	No	Yes	No	No	Yes	No	No
**Active cancer**	No	No	No	No	No	No	Yes	No	No	No	No
**Concomitant proximal DVT**	n.e.	n.e.	Yes	Yes	No	Yes	Yes	No	—	No	No
**Prior pulmonary hypertension**	—	No	No	—	No	No	No	No	No	—	—
**Presentation and severity**											
**Contraindication to lysis**	No	Absolute	No	No	Relative	No	Absolute	No	No	No	No
**Symptom duration, days**	17	17	17	18	20	21	23	24	31	32	33
**SCAI SHOCK stage**	C	A	B	C	B	A	C	A	B	A	B
**ESC risk category**	Interm.-high	High	Interm.-high	Interm.-high	Interm.-high	Interm.-high	High	Interm.-high	Interm.-high	Interm.-high	Interm.-high
**BOVA score**	4	4	5	5	4	4	7	6	—	2	—
**PESI score**	109	137	155	98	—	103	144	—	106	38	76
**sPESI score**	1	2	2	3	—	1	3	4	2	0	1
**Biomarkers and haemodynamics**											
**Troponin I, ng/L**	85	306	1084	3	455	46	588	1700	767	—	—
**Systolic BP, mmHg**	110	121	121	125	135	110	90	100	100	124	100
**Heart rate, bpm**	105	100	140	114	105	95	110	85	105	66	135
**SpO_2, %**	95	97	90	91	93	95	88	86	87	98	92
**RV basal diameter, mm**	—	47	43	—	56	50	50	50	55	—	—
**PASP (echo), mmHg**	—	53	60	—	40	60	60	70	40	—	—
**Invasive systolic PAP, pre (mmHg)**	58	52	46	59	60	60	70	70	47	—	55
**Invasive systolic PAP, post (mmHg)**	51	50	21	55	30	48	48	65	37	—	45
**Procedure**											
**PE location**	Bilat. main	Bilat. main	Bilat. main	Bilat. main	Bilat. main	Lobar/seg.	Bilat. main	Bilat. main	Bilat. main	Unilat. main	Bilat. main
**Device**	Indigo	FlowTriever	FlowTriever	Indigo	FlowTriever	FlowTriever	FlowTriever	FlowTriever	FlowTriever	—	Aspir. cath.
**Procedural success**	Yes	Yes	Yes	—	Yes	Yes	Yes	Yes	Yes	Yes	Yes

BMI, body mass index; DVT, deep vein thrombosis; SCAI SHOCK, Society for Cardiovascular Angiography and Interventions shock classification (A, at risk; B, beginning/compensated [tachycardia without hypoperfusion]; C, classic shock requiring vasoactive support; D, deteriorating; E, extremis/mechanical circulatory support); ESC, European Society of Cardiology; PESI, Pulmonary Embolism Severity Index (sPESI, simplified); BP, blood pressure; SpO_2, peripheral oxygen saturation; RV, right ventricular; PASP, pulmonary artery systolic pressure (echocardiographic); PAP, pulmonary artery pressure (invasive);SCAI stage was assigned as: E if veno-arterial ECMO, D if in-hospital cardiac arrest, C if vasoactive amines were required, B if tachycardia (heart rate >100 bpm) without vasoactive support, otherwise A. Dashes indicate data not recorded in the registry. In the invasive systlic PAP, pre and post precedes refers to the procedure.
